# Galvanin is an electric-field sensor for directed cell migration

**DOI:** 10.1101/2024.09.23.614580

**Published:** 2024-09-24

**Authors:** Nathan M. Belliveau, Matthew J. Footer, Amy Platenkamp, Heonsu Kim, Tara E. Eustis, Julie A. Theriot

**Affiliations:** Department of Biology and Howard Hughes Medical Institute, University of Washington, Seattle, WA 98195, USA

## Abstract

Directed cell migration is critical for the rapid response of immune cells, such as neutrophils, following tissue injury or infection. Endogenous electric fields, generated by the disruption of the transepithelial potential across the skin, help to guide the movement of immune and skin cells toward the wound site. However, the mechanisms by which cells sense these physical cues remain largely unknown. Through a CRISPR-based screen, we identified Galvanin, a previously uncharacterized single-pass transmembrane protein that is required for human neutrophils to change their direction of migration in response to an applied electric field. Our results indicate that Galvanin rapidly relocalizes to the anodal side of a cell on exposure to an electric field, and that the net charge on its extracellular domain is necessary and sufficient to drive this relocalization. The spatial pattern of neutrophil protrusion and retraction changes immediately upon Galvanin relocalization, suggesting that it acts as a direct sensor of the electric field that then transduces spatial information about a cell’s electrical environment to the migratory apparatus. The apparent mechanism of cell steering by sensor relocalization represents a new paradigm for directed cell migration.

## Introduction

Directed cell migration is important for many aspects of biology. In mammals, directed migration drives processes critical to development (1), immune system function (2), and tissue regeneration after injury (3). Much of our understanding of how migrating cells effectively sense and respond to environmental signals comes from work on chemotaxis, mediated by transmembrane receptors that bind specific chemical ligands and transduce those signals to reorient the mechanical force-generating components of the cytoskeleton, and thereby direct persistent directional migration (4, 5). Less is understood about how cells sense and integrate other available spatial information about their physicochemical environment, such as gradients in temperature, pH, or matrix stiffness (6). Directed cell migration in response to acute injury presents a particularly interesting challenge, as the injury must generate a new kind of spatial information directing cells to move toward the wound that was not present in the pre-existing normal structure of the tissue. Here, wound-induced electric fields represent one such directional signal. Nearly all polarized animal epithelia maintain distinct ionic environments on their apical versus basolateral sides due to asymmetric ion transport, and the epithelial barrier presents electrical resistance, normally resulting in a transepithelial potential difference (7, 8). Acute disruption of epithelial barrier integrity by physical injury causes a short-circuit in the transepithelial potential, generating a wound-induced endogenous electric field with magnitudes of 50–500 mV/mm that can persist for many hours (9–11). Many cell types, including epithelial cells and tissue-resident immune cells, have been found to migrate directionally in response to such electrical cues in a process called galvanotaxis (or electrotaxis) (12), which has been proposed to play an integral role in the wound healing response (13).

Although it has been known for well over a century that many motile cell types can perform galvanotaxis, the molecular mechanism of electric field detection remains elusive. To mediate long-distance directed cell migration, a directional signal generated at the cell surface must be transduced to the mechanical elements of the cytoskeleton. Indeed, several intracellular signaling components known to be involved in chemotaxis have also been shown to contribute to galvanotaxis (12, 14, 15), indicating that galvanotaxis is a cell-regulated biological response rather than a purely physical response to forces generated by the influence of electric fields on charged macromolecules. However, due to electrostatic shielding and poor conductance across the plasma membrane, it is unlikely that the relatively weak wound-induced electric fields can directly affect the localization or activity of intracellular signaling components (18, 23–25). While several different kinds of cell surface proteins have been implicated in galvanotaxis, including a voltage-sensitive phosphatase (15), the EGF receptor (16), and several ion channels (17), it is not clear whether any of these candidates act as direct sensors of the electric field. For very rapidly moving cells, biophysical experiments and theoretical modeling have most strongly supported a hypothesis that cells can sense the presence and orientation of electric fields via spatial redistribution of charged cell surface macromolecules due to electrophoresis and/or electroosmosis in the plane of the plasma membrane (18–20). Therefore, we sought to discover cell surface proteins that act directly as electric field sensors.

## Results

**Figure 1.**
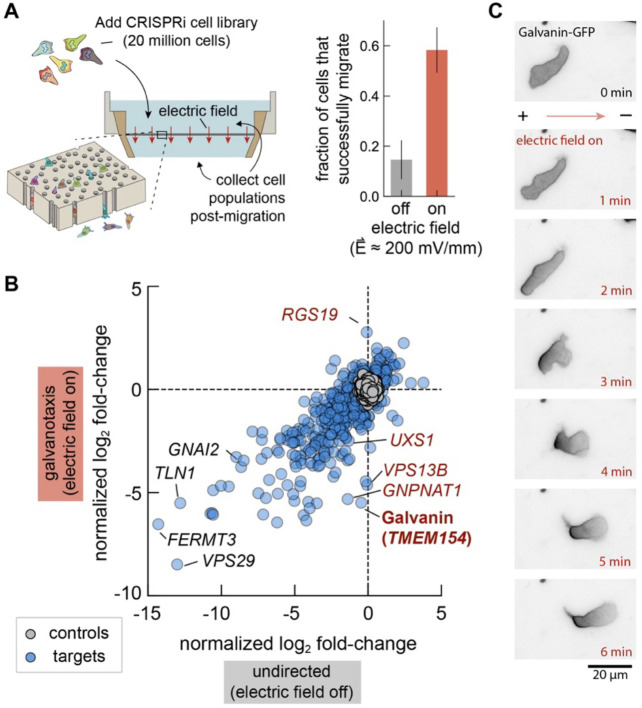
CRISPR interference screen identifies Galvanin as a putative electric field sensor for directed cell migration. **A.** Left: Schematic of galvanotaxis screen. A pooled CRISPRi library of dHL-60 neutrophil-like cells migrate through a membrane with 3 μm diameter pores. Collection of the sub-population that migrate through and, separately, those that remain above the membrane, allow sgRNA target enrichment and gene candidate identification. Right: Fraction of cells collected in the bottom reservoir after two hours. **B.** Summary of focused library screen targeting 1,070 genes for knockdown. Data points show the normalized log_2_ fold-change averaged across three sgRNAs per gene across independent experiments (11 replicates for galvanotaxis and 10 replicates for undirected migration). Control values were generated by randomly selecting groups of three control non-targeting sgRNAs. **C.** Fluorescence micrographs show rapid localization of Galvanin (TMEM154)-GFP toward the anodal pole when differentiated HL-60 neutrophils are exposed to an electric field (300 mV/mm). See also Supplemental Movie 1.

Human neutrophils and neutrophil-like cell lines exhibit impressively rapid directional migration in response to a wide variety of chemical cues (16), and also migrate toward the cathode in an applied electric field (12). The HL-60 cell line, originally derived from a patient with promyelocytic leukemia, can be differentiated in culture to a neutrophil-like cell phenotype (17) and is amenable to genome-wide unbiased genetic screen using CRISPR or CRISPR-interference (CRISPRi) approaches (18, 19). We engineered a device to spatially separate millions of differentiated HL-60 cells based on their capacity to migrate toward the cathode upon exposure to physiologically relevant electric field strengths (Fig. 1A, further detailed in Fig. S1–S2). Cells were added to the top of a membrane containing 3 μm diameter pores and directed downward by applying an electric field with the anodal side above and the cathodal side below the membrane. We could then collect both the cells that successfully migrated through the membrane from the bottom reservoir and those that remained above. Application of an electric field of about 200 mV/mm substantially increased the fraction of cells that could be recovered in the bottom reservoir (Fig. 1B), with approximately 60% of the initial cell population recovered after two hours. In contrast, undirected migration in the same device in the absence of an electric field resulted in recovery of only 15% of the initial cell population in the lower reservoir.

We performed a genome-wide unbiased genetic screen in two phases. Our initial screen was performed using a genome-wide CRISPRi cell library including sgRNAs targeting 18,901 genes, which was used to narrow our focus in a secondary screen targeting only the 1,070 genes whose knockdown showed the strongest effects on cell migration probability. This approach allowed us to increase the coverage per sgRNA in our secondary screen, and thereby improve our statistical confidence for the effects of individual gene candidates (see Supplemental Methods). To better distinguish between perturbations specific to sensing the electric field versus those affecting cell migration more generally, we compared results from two experimental conditions: a) exposure of cells to an electric field (200 mV/mm) and b) undirected cell migration in the same device in the absence of an electric field. In the secondary screen, we found 473 genes where CRISPRi knockdown significantly disrupted migration in the presence of an electric field and 544 genes whose knockdown significantly affected undirected migration, relative to control sgRNAs present in the library (Fig. 1B; adjusted p-value with cutoff of 0.05). The majority of these candidate genes had significant effects in both conditions (Pearson correlation *r* = 0.8 between normalized log_2_ fold-change values), reflecting the commonality of migration machinery necessary during neutrophil migration regardless of the nature of the external cue (18, 20, 21). A smaller subset of 111 genes, however, were identified as significantly altering cell migration probability only in the presence of the electric field. This subset included *TMEM154* (transmembrane protein 154), that we refer to as Galvanin, which stood out as the transmembrane protein whose knockdown gave the strongest electric-field-specific phenotype.

Galvanin is predicted to be a single-pass transmembrane protein (161 amino acids, UniProt ID: Q6P9G4). In primary neutrophils, its transcriptional levels are roughly similar to other integral membrane proteins such as the LPS receptor CD14 and the integrin ɑ_M_ (22). We confirmed that Galvanin localizes to the HL-60 cell plasma membrane by tagging the intracellular C-terminus with eGFP. As a putative electric field-sensitive protein, we hypothesized that its localization would become spatially biased when cells were exposed to an electric field. Using an agarose overlay to confine migrating cells to a single plane on a glass coverslip, we exposed the cells to an electric field of 300 mV/mm. We indeed observed relocalization of Galvanin-GFP, which rapidly becomes biased to the anodal (positive) pole of the cell within about one minute of exposure (Fig. 1C, Supplemental Movie 1). For these cells, which migrate toward the cathode, this equates to relocalization of the protein to the cell rear and is consistent with electrophoresis of a net negatively charged protein. From the amino acid sequence alone, the ectodomain is expected to have a net charge of −7*e*, which is too low to account for such a notably biased distribution (23). One likely explanation for this disparity is glycosylation of the ectodomain, which could substantially increase its negative charge. Protein glycosylation, referring to the addition of carbohydrates to the polypeptide following translation, has been implicated in galvanotaxis of other cell types (18, 19, 33). These modifications often form branched oligosaccharide chains that terminate in negatively charged sialic acid groups and can confer a strongly negative net charge on glycosylated proteins at neutral pH (24). Using NetNGlyc 1.0 (35) and NetOGlyc 4.0 (36), we found six amino acid positions in the ectodomain of Galvanin that may contain glycosylation (one N-type and up to five O-type modifications).

We followed up on several of the screen candidate genes to better assess how the genetic perturbations altered cell migration. In addition to Galvanin itself, we focused on three other candidate genes that might be expected to affect the cell surface presentation of a putative electric field sensor: VPS13B which is involved in membrane trafficking, and GNPNAT1 and UXS1 which are both enzymes involved in protein glycosylation. We generated individual stable HL-60 knockdown lines for each of these four candidates, then differentiated them and embedded them in three-dimensional collagen gels, where their speeds and migration directions were measured using video microscopy. Consistent with our screen results, each cell line appeared to migrate normally in the absence of an electric field. Importantly, in the presence of an electric field, each cell line showed reduced directionality when compared to migration of cells with a non-targeting sgRNA (Fig. S2E), supporting their involvement in the directional response.

**Figure 2.**
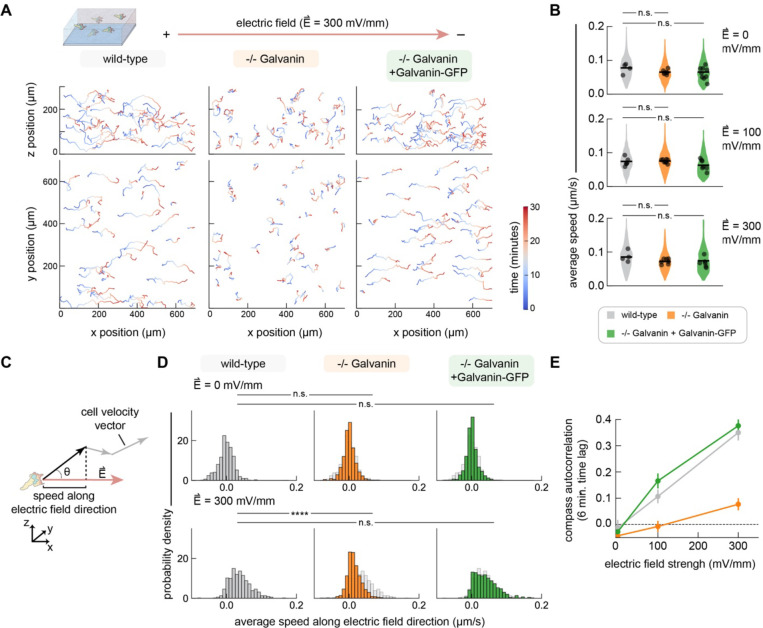
Galvanin is critical for persistent cathodal migration in HL-60 neutrophils. **A.** Nuclear tracking was performed during migration of HL-60 neutrophils in a collagen gel exposed to an electric field (300 mV/mm). Data represents a subset of data, with only 50 cell tracks shown per cell line (dHL-60 neutrophil wild-type, −/− Galvanin knockout, and the genetic rescue with Galvanin-GFP). **B.** Average speed calculated based on nuclear tracking with 3 minute time intervals. Individual data points represent average values across cells from a single field of view. The shaded regions show a kernel density estimation of the distribution of measurements across all fields of view (two-sided Mann-Whitney U test found no significant difference). **C.** Schematic showing calculation of directed speed along the electric field vector. **D.** Histograms summarize average speed along the electric field direction. Individual measurements represent the average speed per cell along electric field direction, with 3 minute tracking intervals (400–1,000 cells and 4,000–9,500 tracking intervals per condition). Wild-type histograms (light gray) are plotted along with the −/− Galvanin knockout and genetic rescue data for reference (p-value < 0.0001, two-sided Mann-Whitney U test). **E.** Longer-term directed movement was determined by defining a compass autocorrelation. For individual cell tracks, the angle *θ* between the cell vector and the electric field vector was determined for each 3 minute time interval. Autocorrelation analysis was performed on the set of corresponding cosine *θ* values and averaged across cells (see *Methods*). Autocorrelation values for a six minute time lag are shown here, with all time lags shown in Supplemental Figure 2. Error bars represent standard error of the mean. For each cell type in **B**, **D**, and **E,** 400–1,000 cells were analyzed per condition (approximately 30–150 cells quantified per imaging acquisition, with 4–9 acquisitions per cell line and per electric field strength).

To better assess the functional contribution of Galvanin to directional migration, we used CRISPR-Cas9 to create two clonal knockout cell lines where the endogenous Galvanin gene was disrupted (Fig. S3Ai). We then assessed their directional migration in collagen gels when exposed to electric fields of varying strength. In Fig. 2A we show tracks of individual migrating cells exposed to a 300 mV/mm field, tracking cell nuclei over a 30-minute period. We observed a notable loss of directed migration in each Galvanin knockout cell line relative to wild-type cells (Fig. 2A middle, Fig. S3Aii). Importantly, by expressing our Galvanin-GFP construct in the Galvanin knockout cell line, we were able to rescue the cathodal directional migration of wild-type cells (Fig. 2A right), demonstrating that Galvanin is necessary for the normal electric field response. Notably, the Galvanin knockout cells were still highly migratory, with average migration speeds indistinguishable from wild-type cells in the presence or absence of an electric field (Fig. 2B); that is, their phenotypic defect lies strictly in their ability to orient toward the cathode and not in any other aspect of cell migration. We further quantified the average speed of individual cells projected along the electric field vector (that is, the electric field-directed component of the cell speed) (Fig. 2C). Plotting the average speeds across all cells tracked, we find a significant loss of directionality in our knockout cell line compared to the wild-type and Galvanin rescue cell lines (Fig. 2D).

We also assessed the directional movement at longer length scales. For migration in a physically complex environment such as fibrous collagen gel, which results in somewhat tortuous cell trajectories, we chose a metric to assess the overall directional bias under a sustained cue. Specifically, we quantified the autocorrelation of measured cosine of the angles (cos *θ*) between the migration vector of individual cells and the electric field vector, which we refer to as the compass autocorrelation (Fig. S3B). This metric remains high when cells move over long periods of time in the same net direction, even if the local path persistence is relatively low (as is typical for rapidly moving neutrophils). Cells exhibited a compass autocorrelation that was dependent on the strength of the electric field and was notably reduced with the Galvanin knockdown cell lines (Fig. S3C). In Fig. 2E we plot the compass autocorrelation at a six-minute time lag, with the Galvanin knockout cell line showing a substantial loss of directional movement along the electric field vector, and complete rescue by Galvanin-GFP. We conclude that Galvanin is an indispensable element for directed migration during galvanotaxis of these rapidly moving neutrophil-like cells.

**Figure 3.**
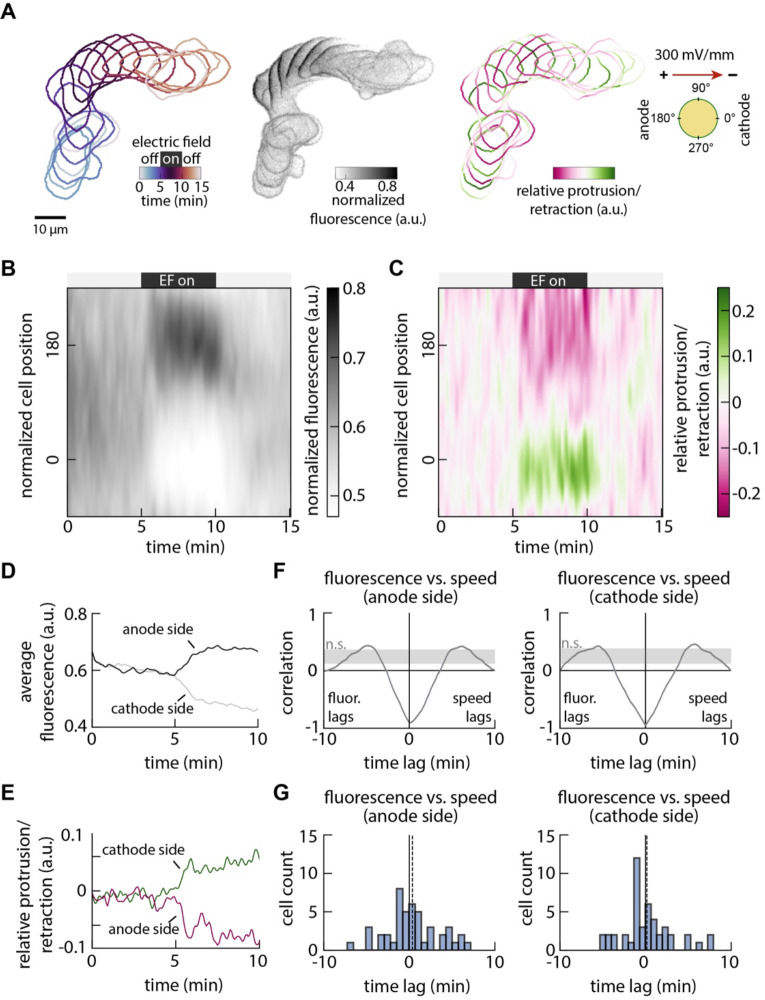
Localization of Galvanin coincides with reduced local membrane speed and front-rear polarization. **A.** Example analysis showing the temporal movement, Galvanin-GFP fluorescence intensity, and cellular protrusion/retraction activity of a single cell. Imaging was performed every 5 seconds over 15 minutes, with cells exposed to an electric field (300 mV/mm) between 5 minutes and 10 minutes. One-minute intervals are overlaid. See also Supplemental Movie 2. **B.** Kymograph shows the normalized Galvanin-GFP intensity, quantified at the cell periphery (in a band of 2 μm thickness) and averaged across 46 cells. Cell contours are defined with 360 positions, with position 0 corresponding to the right-most position relative to the cell centroid. **C.** Kymograph quantifies membrane speed along the cell periphery, by comparing cell shape changes between 5 second intervals and correcting against bulk cell translocation. Values represent an average across the 46 cells considered in part B. **D.** Averaged fluorescence at the anodal side (positions +120 to +240) and cathodal side (positions −60 to +60) during the first 10 minutes. **E.** Averaged membrane speed at the anodal side (positions +120 to +240) and cathodal side (positions −60 to +60) during the first 10 minutes. **F.** Cross-correlation analyses comparing the averaged fluorescence and membrane speeds (parts D-E) at the anodal and cathodal sides of the cell. Dash lines indicate the average minimum lag (0.1 minutes for the anode side and 0 minutes for the cathode side). The shaded region represents correlation values that are below a 99% confidence interval (see *Methods*). **G.** Cross-correlation analysis was performed using the Galvanin-GFP fluorescence and membrane velocities averaged across all 46 individual cells. Histograms show the lag corresponding to the minimum correlation value. Dashed lines near zero indicate the average lag value (anode side: 0.4 minutes; cathode side: 0.2 minutes).

To better understand how Galvanin supports directed migration, we quantified the dynamics of its relocalization on the membrane while also measuring changes in local protrusion and retraction activity and front-rear cell polarization (25, 26). Using our under-agarose assay to confine cells to migrate in a single plane, we monitored cell migration at high spatial and temporal resolution during five minutes of undirected migration, five minutes of exposure to an electrical stimulus of 300 mV/mm, and five minutes of recovery (Fig. 3A). Galvanin-GFP localization was measured around the periphery of individual cells, and cell migration changes were quantified using the Adapt package (27), which measures the relative local protrusion or retraction of the cell edge, as a function of position around the cell perimeter. This analysis was performed on 46 cells, enabling us to generate population-averaged kymographs of Galvanin localization dynamics (Fig. 3B) and an associated mapping of protrusion and retraction (Fig. 3C). We found that the cellular response to the electric field is almost immediate, with a relocalization of Galvanin-GFP to the anode that was nearly complete within about one minute (Fig. 3D). Accompanying this was a notable change in the spatial distribution of protrusion and retraction, with an immediate increase in retraction at the newly forming cell rear and an increase in protrusion at what becomes the cell front (Fig. 3E). To compare the timing of the Galvanin-GFP relocalization with the changes in protrusion and retraction activity, we performed a cross-correlation to compare these two parameters over time (Fig. 3F). On both the rear (anodal) and front (cathodal) sides of the cell, the Galvanin-GFP relocalization was tightly coupled to the protrusion/retraction activity, with a maximum negative correlation at zero time lag, indicating that the two processes are nearly simultaneous (within the time resolution of these experiments). This finding was also maintained when measuring the cross-correlation between Galvanin-GFP signal and protrusion/retraction activity for individual cells (Fig. 3F). These results suggest that Galvanin relocalization defines the cell front and cell rear for directed cell migration during exposure to an electrical cue, either by locally activating retraction at the rear, or by removing inhibition of protrusion at the front.

**Figure 4.**
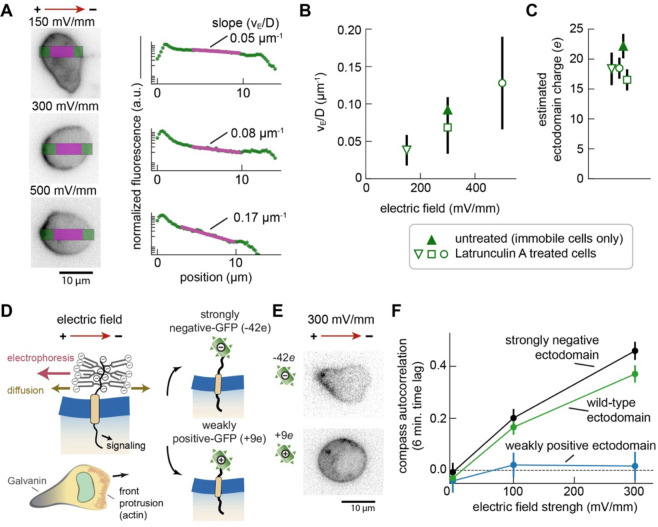
The sensory mechanism of Galvanin depends on a highly charged ectodomain. **A.** Left: Example micrographs of Galvanin-GFP in individual cells exposed to an electric field for 10 minutes. Cells were treated with Latrunculin A to immobilize them following initial migration under agarose (see *Methods*). The intensity profile along the electric field vector direction was quantified to estimate the slope in the decay in fluorescence intensity, expected to be proportional to v_E_/D. Right: semilogarithmic plots of corresponding fluorescence profiles for the examples shown, with green indicating the entire cell width, and magenta corresponding to the fit region, selected to avoid the more intense cell periphery. The estimated v_E_/D values are indicated for each fit. **B.** Summary of v_E_/D values across the different electric field strengths. Error bars indicate standard deviation across 13 cells (150 mV/mm), 23 cells (300 mV/mm), and 26 cells (500 mV/mm). Analysis of non-motile cells present in the experiments of Figure 3 are also included (4 cells). **C.** Estimated ectodomain charge based on the v_E_/D values of part B (see *Methods*). Error bars indicate standard error of the mean. **D.** Left: schematic illustrating our model of Galvanin electrophoresis due to the net negative charge. Branched changes represent expected glycosylation of ectodomain based on 6 predicted O-/N- type modifications. Right: the ectodomain was altered using engineered GFP proteins with either a strongly negative charge (−42*e* net ectodomain) or weak charge (+9*e* net ectodomain charge). **E.** Representative fluorescence micrographs of cells expressing the engineered Galvanin constructs exposed to a 300 mV/mm electric field (imaging performed over 2–3 data; −42*e,* n=40 cells; +9*e*, n=31 cells). The strongly negative construct shows localization to the anodal side of the cell, similar to the wild-type Galvanin-GFP, while the weakly positive construct remains uniformly distributed. **F.** Compass autocorrelation of cells migrating in a collagen gel. The −/− Galvanin knockout cells expressed Galvanin-GFP (green), −42GFP-Galvanin (black), or +9GFP-Galvanin (blue). Autocorrelation analysis on the cosine *θ* between cell trajectories and the electric field vector. Data points represent average across analysis performed on individual cell tracks (180–750 cells per cell line and per electric field strength); error bars: standard deviation of the mean.

In theory, the rapid anode-directed electrophoresis of Galvanin should depend on the Coulombic interaction between the charged ectodomain of the protein and the electric field, with the field unable to penetrate into the intracellular space (28–30). The stable spatial distribution of Galvanin-GFP we observed after several minutes of electric field exposure should represent a balance between the Coulombic movement toward the positive, anodal pole with electrophoretic velocity *v*_*E*_ and equilibration by diffusion with an effective diffusion coefficient D (23). Specifically, we expect the steady-state Galvanin concentration to vary along the electric field vector with an approximate exponential decay (31), with a characteristic length that depends on the ratio *v*_*E*_/D. This ratio can be used to infer the net charge on the protein (see Supplemental Methods). To more precisely quantify this ratio and avoid confounding factors introduced by migrating cells (including possible membrane flow), we repeated the under-agarose experiments in the presence of Latrunculin A. This drug inhibits filamentous actin assembly and allows us to immobilize cells as they begin to migrate under the agarose overlay, maintaining their flattened shape against the coverslip. Latrunculin A-treated cells were exposed to electric field strengths of 150 mV/mm, 300 mV/mm, and 500 mV/mm (Fig. 4A). The slope of the fluorescence intensity profiles, plotted on a semilogarithmic scale, were then used to estimate the ratio *v*_*E*_/D at each electric field strength. As shown in Fig. 4B, the *v*_*E*_/D ratio increased from about 0.05 μm^−1^ to 0.2 μm^−1^ over this range. The net charge is proportional to the *v*_*E*_/D ratio, normalized by the electric field strength, and strikingly, we arrive at a similar estimate of the net charge on Galvanin across all conditions, with a value of −18*e* (±1.1 SEM) averaged across all the data (Fig. 4C). Using this same dataset, we were also able to estimate the effective diffusion coefficient D for Galvanin-GFP by quantifying its equilibration back to a uniform distribution after the electric field stimulus was removed (Fig. S4A–D), obtaining a value for D of 0.53 μm^2^/s (±0.1 SEM), which is consistent with expectations for a single-pass transmembrane protein (32). In summary, its high mobility and high net negative charge enable Galvanin to exhibit rapid dynamics as an electric field sensor.

Due to the limited structural characterization of Galvanin, it remained unclear whether additional features of the protein might be important for its sensory function, or whether the net charge of the ectodomain was by itself sufficient. To test this, we removed the wild-type ectodomain and replaced it with engineered protein domains that have a known net negative charge (Fig. 4D). We utilized previously developed “super-charged” GFP proteins (33). We hypothesized that a highly negatively charged engineered ectodomain would rescue directed migration in our Galvanin knockout cell line, assuming net charge is the sole critical factor. We were able to express a highly negative construct, replacing Galvanin’s ectodomain with a negatively charged XTEN linker domain (34) and negatively charged version of GFP, yielding an expected total net charge of −42*e* at pH 7. Additionally, we expressed a construct where the ectodomain was replaced with a weakly positive GFP, having an expected net charge of +9*e* at pH 7. Although these mutated forms of GFP exhibited poorer fluorescence, with the +9*e* form showing more variable membrane and cytosolic fluorescence, both constructs still localized to the plasma membrane in Galvanin knockout cells (Fig. 4E).

We assessed the functional activity of these engineered constructs by expressing them in Galvanin knockout cells and exposing the cells to an electric field. Using Latrunculin A as noted above to immobilize cells, the −42*e* construct showed a strong anodal localization (Fig. 4E). Estimating v_E_/D as described above, we calculate a net charge to be 37e (±2.5 SEM), which is close to the calculated value and gives us confidence in the charge measurement technique. In contrast, the +9*e* construct showed no notable change in GFP distribution when cells were exposed to an electric field, suggesting that the weaker charge is insufficient for relocalization at these electric field strengths, as had been previously predicted based on theoretical considerations (23). To assess migration in cell lines expressing these engineered constructs, we turned to our cell tracking assay in collagen. Importantly, the −42*e* construct was able to completely rescue directed cell migration during exposure to an electric field, with even a slightly higher compass autocorrelation than wild-type cells (Fig. 4F). At 100 mV/mm, the −42*e* construct also led to an increased average speed along the electric field direction when compared to the Galvanin-GFP rescue cell line (Fig. S4E). In contrast, and consistent with the lack of spatial relocalization of the +9*e* construct, the weakly charged ectodomain was unable to rescue the directed migration response. In summary, we find that the high net negative charge of the ectodomain is sufficient to mediate the sensory response of Galvanin.

## Discussion

We have identified Galvanin, a previously uncharacterized membrane protein, that acts as an electric field sensor and mediates cathode-directed cell migration for human neutrophils. While past work has demonstrated that many plasma membrane proteins undergo electrophoresis across cells exposed to an electric field (23, 35–39), it has been challenging to connect bulk protein electrophoresis to galvanotaxis or to directed migration at the whole-cell level (40). Our demonstration that electrophoretic relocalization of a single protein based on the net charge of the ectodomain is necessary and sufficient to reorient directed cell migration represents the first description of an electric field sensor of this class. Galvanin’s electrophoretic response is rapid, enabling migrating cells to quickly repolarize within minutes of exposure to an electric field. For the rapidly migrating neutrophil cell type considered here, this time scale is consistent with the rapid response at sites of tissue injury. In this context, the wound-induced electric field would provide an additional layer of directional information, alongside other well-characterized chemical guidance cues involved in wound repair (13).

In neutrophils, Galvanin accumulates at the anodal side, which becomes the cell rear. The close coupling we observe between Galvanin relocalization and changes in local cell protrusion and retraction behavior suggests that Galvanin may steer cell migration either by locally activating retraction (for example, by stimulating myosin II contractility) or by locally inhibiting protrusion (for example, by inhibiting activity of the Arp2/3 complex responsible for branched actin network growth). In canonical neutrophil chemotaxis, chemoattractant receptors (typically GPCRs) are uniformly distributed on the cell’s plasma membrane, and ligand occupancy of the receptors is higher on the side of the cell closer to the source of the chemoattractant because of its concentration gradient (16, 41). Downstream signaling from the activated GPCRs leads both to stimulation of actin network assembly at the presumptive cell front and to stimulation of myosin II-based contractility at the cell rear (42, 43). Because of the strong positive feedback within the respective “frontness” and “backness” mechanochemical modules, and strong negative feeback between them (44–46), as well as the mechanical influence of tension in the cell plasma membrane to enforce tight and near-immediate coupling of changes in protrusion at one end of the neutrophil to drive retraction on the opposite side and vice versa (47, 48), local activation of retraction or local inhibition of protrusion by Galvanin could each be completely sufficient to reorient whole-cell polarity for directed migration of neutrophils. Future work will be needed to understand how Galvanin interacts with and regulates actomyosin activity in migrating neutrophils, and whether it utilizes signaling pathways that have been implicated in galvanotaxis of other cell types (12, 15, 23, 49, 50).

Beyond the neutrophil-like cell type considered here, it will be interesting to see how Galvanin behaves in other cell types. In humans, transcriptional data suggests highest expression in immune cells and skin (GeneCards ID: GC04M152618), in line with the perceived importance of galvanotaxis during wound healing and suggesting further work in this context. The ability for cells to alter Galvanin’s net charge through glycosylation, which tends to be cell-type specific (51, 52), also offers an interesting strategy cells could employ to vary its functional activity. In the work performed here, our ability to engineer Galvanin and alter its physical and electrical properties demonstrates a tunable biological system that can be used to provide control over directed cell movement.

## Supplementary Material

Supplement 1

Supplement 2

Supplement 3

Supplement 4

## Data Availability

Sequence data generated from CRISPRi screens and RNA-seq are uploaded to the Sequence Read Archive and can be accessed under BioProject accession code PRJNA1147270 (https://www.ncbi.nlm.nih.gov/bioproject/PRJNA1147270). All data files used to generate figures are available in our GitHub repository, noted in the Code Availability section below. Due to the large size of raw image files used for cell tracking, these are not included but are available from the corresponding authors upon request. Source data are provided with this paper. Processed data, code, and figure generation scripts generated with the Python package matplotlib (v. 3.5.2) are publicly available as a GitHub repository (nbellive/CRISPRi_galvanotaxis_pub).
